# Pregnant adolescents living with HIV: what we know, what we need to know, where we need to go

**DOI:** 10.7448/IAS.20.1.21858

**Published:** 2017-08-04

**Authors:** Tegan Callahan, Surbhi Modi, Jennifer Swanson, Bernadette Ng’eno, Laura N. Broyles

**Affiliations:** ^a^ Division of Global HIV and Tuberculosis, U.S. Centers for Disease Control and Prevention (CDC), Atlanta, GA, USA; ^b^ Rollins School of Public Health, Hubert Department of Global Health, Emory University, Atlanta, GA, USA

**Keywords:** Pregnant adolescents, HIV, antenatal care, infant outcomes, PMTCT cascade, retention

## Abstract

**Introduction**: HIV-infected pregnant and breastfeeding adolescents are a particularly vulnerable group that require special attention and enhanced support to achieve optimal maternal and infant outcomes. The objective of this paper is to review published evidence about antenatal care (ANC) service delivery and outcomes for HIV-infected pregnant adolescents in low-income country settings, identify gaps in knowledge and programme services and highlight the way forward to improve clinical outcomes of this vulnerable group.

**Discussion**: Emerging data from programmes in sub-Saharan Africa highlight that HIV-infected pregnant adolescents have poorer prevention of mother-to-child HIV transmission (PMTCT) service outcomes, including lower PMTCT service uptake, compared to HIV-infected pregnant adults. In addition, the limited evidence available suggests that there may be higher rates of mother-to-child HIV transmission among infants of HIV-infected pregnant adolescents.

**Conclusions**: While the reasons for the inferior outcomes among adolescents in ANC need to be further explored and addressed, there is sufficient evidence that immediate operational changes are needed to address the unique needs of this population. Such changes could include integration of adolescent-friendly services into PMTCT settings or targeting HIV-infected pregnant adolescents with enhanced retention and follow-up activities.

## Introduction

Over the past three years, there has been an increase in global resources and advocacy targeting HIV prevention, care and treatment for adolescents worldwide. Through a public–private partnership, the US President’s Emergency Plan for AIDS Relief (PEPFAR) launched the DREAMS Initiative in 2014 to reduce new HIV infections among adolescent girls and young women in 10 sub-Saharan African countries [1]. Similarly, the Joint United Nations Programme on HIV/AIDS (UNAIDS) and The United Nations Children’s Fund (UNICEF) announced in July 2014 the All In Initiative, aiming to ensure that adolescents infected and affected by HIV are adequately included in the global HIV response [2].

Although these global initiatives have focused energy and resources on the large and vulnerable population of adolescents within sub-Saharan Africa, special attention is needed for a subgroup of this population - the pregnant and breastfeeding adolescents living with HIV. Prevention of mother-to-child HIV transmission (PMTCT) programmes must join the global momentum to focus on the needs of HIV-infected pregnant adolescent girls aged 10–19 years and develop service delivery packages which address their needs as both HIV-infected adolescents *and* HIV-infected mothers.

### A plurality of risks for adolescent women in sub-Saharan Africa

In sub-Saharan Africa, 30% of all new HIV infections occur in young women under the age of 25 [3]. This vulnerability is compounded by the fact that more than 50% of births in sub-Saharan Africa take place during adolescence, particularly between 15 and 19 years [4]. This risk environment is further complicated by the developmental and biological changes of adolescence, such as sexual debut, and additional risk behaviours such as older and concurrent sexual partners and inconsistent contraception use [5–7]. These experiences and behaviours result in an increased risk for both HIV infection and pregnancy during adolescence [8]. Given the plurality of risks faced by adolescents and young women in sub-Saharan Africa, HIV prevention programming and clinical services should address the HIV-related education and clinical service needs as well as sexual and reproductive health needs unique to adolescents.

### Differential outcomes for adolescent pregnant girls along the PMTCT cascade

Data on adolescents living with HIV in sub-Saharan Africa have highlighted differential outcomes for adolescents compared to adults across the continuum of HIV prevention, care and treatment services, frequently referred to as the HIV “cascade”. National survey data from across sub-Saharan Africa show that less than a third of adolescents age 15–19 have ever received an HIV test [5]. Adolescents living with HIV are also reported to have lower antiretroviral therapy (ART) adherence and virologic suppression [9,10]. Recent population-based surveys in three African countries (Malawi, Zambia and Zimbabwe) found that community viral load suppression among HIV-infected adolescents and young people 15–24 years was 42% compared to 63% among adults [11]. Emerging data on the outcomes of HIV-infected pregnant adolescents indicate similar differential outcomes along the PMTCT-specific service cascade as well. This evidence highlights the need for an urgent focus on HIV prevention and treatment activities for this age group in order to improve health and reduce HIV transmission to infants and sexual partners. The next section summarizes the current evidence on pregnant adolescents within the PMTCT cascade, including HIV testing in antenatal care (ANC), uptake of maternal HIV-related services, retention in care and service uptake for their HIV-exposed infants.

## Discussion

### HIV testing

Recent work from West Africa has highlighted lower HIV testing rates across the region among adolescents (10–19 years) attending ANC compared to their older counterparts [12]. A review of demographic and health surveys and multiple indicator cluster surveys data showed statistically significant (*p* < 0.05) gaps in HIV testing for adolescent (10–19 years) compared to older (≥20 years) ANC clients in 12 out of the 21 Western and Central African countries where data were available [12]. These results are consistent with data drawn from Joint United Nations Programme on HIV/AIDS HIV estimates, nationally representative household surveys, behavioural surveillance surveys and published literature showing low HIV testing uptake among adolescents in general [5]. In contrast, a nationally representative, cross-sectional study in Kenya found no difference in HIV testing rates in the ANC setting between adolescents <19 years (95.7%) and adult women (91.6%) (odds ratio (OR) 0.95 (95% confidence interval (CI) 0.19–4.71), *p* = 0.95) [13]. These investigators, however, found that fewer HIV-infected pregnant adolescents were already on ART for their own health prior to entry into ANC (4.8% of HIV-infected pregnant adolescents compared to 43.1% of adult women) [13]. These findings highlight the importance of ANC services as an opportunity to identify HIV-infected pregnant adolescents and rapidly initiate ART to prevent mother-to-child HIV transmission and decrease maternal morbidity and mortality. ANC is also an opportunity to identify HIV-negative adolescents with risk behaviours who may benefit from targeted HIV prevention interventions.

### Linkage and uptake of PMTCT services

The currently available evidence demonstrates lower PMTCT service uptake for HIV-infected pregnant and breastfeeding adolescents when compared to adult mothers. Data from South Africa showed that a significantly lower proportion of adolescent mothers had a CD4 cell count taken during pregnancy (66.5% adolescents, 81% adult women, *p* < 0.0001) and obtained the results (75.7% adolescents, 84.4% adults, *p* < 0.0001) [14]. An earlier observational cohort of HIV-infected pregnant and breastfeeding women in Tanzania demonstrated that among those who were ineligible to start ART based on CD4 criteria and offered antiretroviral (ARV) prophylaxis, young age was independently associated with an increased risk of not starting ARV prophylaxis before delivery. Among mothers less than 24 years old, 61% declined to start ARV prophylaxis compared to 18% of adult mothers [15]. These findings are similar to those from a recent study in Kenya where a significantly lower proportion of adolescents who were not on ART pre-pregnancy took ARVs to reduce mother-to-child transmission of HIV compared to adult women not previously on ART (65.0% vs. 85.8%, respectively (*p* = 0.01)) [16]. The global policy shift to immediate and lifelong ART initiation for all pregnant and breastfeeding women living with HIV (“Option B+”) mitigates the need to offer ARVs for prophylaxis during pregnancy and breastfeeding. Harmonized and simplified treatment regimens for all people living with HIV, including pregnant adolescents and women, as part of the World Health Organization “Treat All” approach along with efforts to raise community awareness of the importance of early HIV treatment, may promote ART uptake for this population [17].

### Retention throughout the PMTCT cascade and HIV-exposed infant outcomes

While limited, current evidence suggests that HIV-exposed infants of adolescent mothers have poorer HIV-related clinical outcomes compared to infants of older women. One study found that a higher proportion of infants of adolescent mothers were infected with HIV compared to infants of adult mothers (10.8% vs. 6.1%, OR 1.7, 95% CI 1.2–2.4) [14], likely reflecting the suboptimal service uptake, adherence and virologic suppression seen in adolescent populations [18].

### Tailoring services to the needs of adolescents

The current evidence suggests that “business as usual” within the PMTCT service cascade has not worked well for adolescent mothers. PMTCT programmes should recognize this population as a vulnerable subgroup and develop evidence-based, enhanced support strategies which address their needs. Early formative research with pregnant adolescents documents concerns about breaches of confidentiality, prejudicial community attitudes towards pregnancy and HIV status and poor interactions between the patient and healthcare provider [19]. “Adolescent-friendly PMTCT services” that emphasize confidentiality, address stigma related to HIV infection and adolescent pregnancy and integrate innovative models of increasing social support should be provided. There is currently limited evidence in the published literature describing support interventions for HIV-infected adolescent mothers, but the available data suggest that targeted psychosocial and family support interventions within the clinical or community settings may be needed to address the barriers to care experienced by HIV-infected adolescent mothers. One study from South Africa showed higher uptake of prenatal care and PMTCT interventions among married HIV-infected adolescent mothers compared to unmarried adolescent mothers living with HIV, demonstrating the importance of social support, and, in particular, partner involvement, throughout the PMTCT cascade [20]. Pregnant HIV-infected adolescents may also be considered a priority population for access to intensive retention interventions such as patient navigators accompanying them through the cascade of services to ensure that barriers are effectively resolved, patient empowerment messages and cash transfer programming.

In addition to prioritization and service delivery adjustments for HIV-infected adolescent mothers, a research agenda is needed to further understand this under-investigated population. The agenda should include formative research to better understand the unique barriers and facilitators of PMTCT service uptake among pregnant adolescent women, assessments of “adolescent-friendly PMTCT” service models to understand their potential impact on clinical outcomes and evaluations of the unique needs of sub-populations of adolescents (e.g. urban versus rural, married versus unmarried, adolescents who engage in transactional sex and adolescents with and without a stable partner).

## Conclusions

From the limited evidence available, we know pregnant adolescents living with HIV and their infants have inferior outcomes along the PMTCT services cascade compared to older mothers and their infants. While we still need to better understand the drivers of these inferior outcomes, clues from the current literature and from programmatic experience justify immediate operational changes which prioritize this vulnerable population and address their unique needs. Such changes could include integration of adolescent-friendly services into PMTCT settings or targeting pregnant adolescents with intensive retention and follow-up activities.

Targeted programming and strategies for adolescent mothers living with HIV will be essential to reaching global goals of controlling the HIV epidemic. Given the high prevalence of both HIV infection and pregnancy during the adolescent period, a focus on optimizing PMTCT services for pregnant and breastfeeding adolescents will be critical to achieving elimination of mother-to-child transmission goals and ensuring HIV-infected adolescents and their families are able to live long, productive lives.
